# Supplementary figures and images for: CNVs Associated with Different Clinical Phenotypes of Psoriasis and Anti-TNF-Induced Palmoplantar Pustulosis
Source: J Pers Med. 2022 Sep 4;12(9):1452. doi: 10.3390/jpm12091452 (PMC9506507; doi:10.3390/jpm12091452)

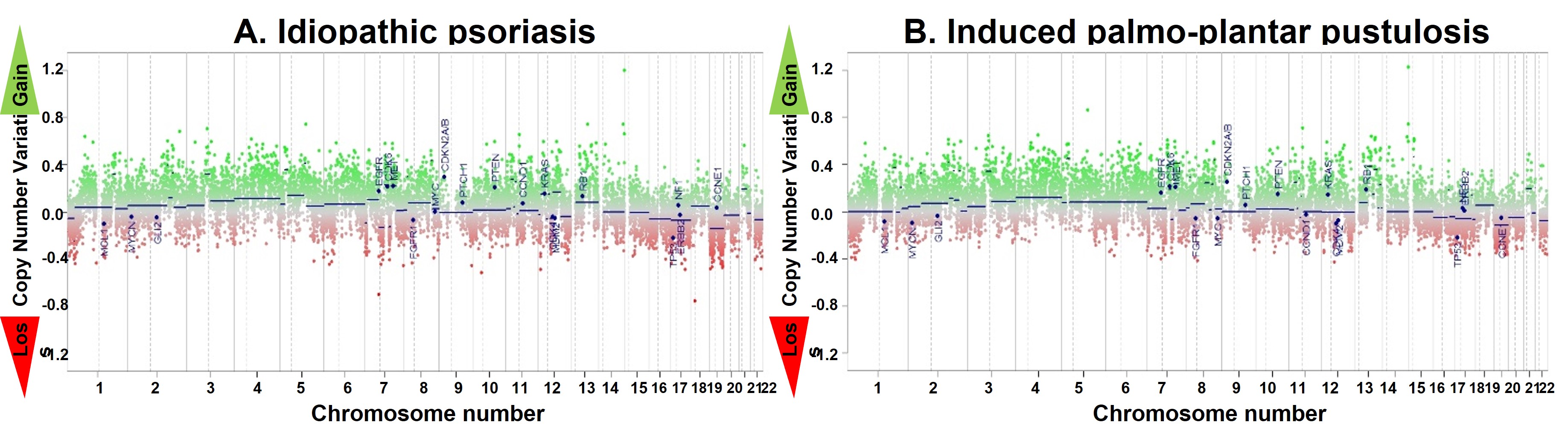

Supplement: Supplementary file 1 [file jpm-12-01452-s001.zip › jpm-1871271-Figure S1.jpg]
